# Laboratory analysis of Au–Pd bimetallic nanoparticles synthesized with *Citrus limon* leaf extract and its efficacy on mosquito larvae and non-target organisms

**DOI:** 10.1038/s41598-020-78662-y

**Published:** 2020-12-10

**Authors:** Savy Panamkuttiyiel Minal, Soam Prakash

**Affiliations:** Advance Parasitology and Vector Control Nano-Biotechnology Laboratory, Department of Zoology, Dayalbagh Educational Institute (Deemed University), Agra, 282005 India

**Keywords:** Environmental sciences, Health care, Risk factors, Materials science, Nanoscience and technology

## Abstract

The current study provides novel results on the synthesis of bimetallic nanoparticles (BNPs) of gold and palladium (Au–Pd) with an eco-friendly and non-toxic aqueous leaf extract of plant *Citrus limon*. The BNPs were characterized and toxicity bioassay was examined on the larvae of the pathogen vectors such as *Anopheles stephensi* and *Aedes aegypti* mosquitoes. The predation efficiency test was evaluated on the invertebrate non-target organisms such as natural predatory nymphs of dragonfly and damselfly. The results of material characterization using UV VIS spectroscopy confirmed the synthesis of Au–Pd BNPs with the appearance of the SPR bands. FT-IR spectroscopy indicates the presence of functional groups containing high amounts of nitro compounds and amines on the surface of BNPs. TEM result shows the presence of spherical polydisperse Au–Pd BNPs in the sample. The XRD pattern displayed the semi-crystalline nature and the changes in the hydrodynamic size and surface potential was determined for the sample at 0 h, 24 h, 48 h, and 72 h of synthesis through DLS and ZP analysis. Au–Pd BNPs Bioassay provided the effective lethal concentrations (LC_50_) against the I–IV instar larvae of *An. stephensi* and *Ae. aegypti* after 24 h, 48 h, and 72 h of exposure. The LC_50_ obtained from the larvicidal bioassay was used to test its effect on the predation efficiency of the selected nymphs which showed increased predation from 40 to 48 h of exposure as compared to the negative control. Hereby, we conclude that Au–Pd BNPs bioassay shows toxic mosquito larvicidal activity at the selected concentration with no lethal effect on the predation efficiency of the selected stage of the predatory non-target aquatic invertebrate insects.

## Introduction

The current COVID-19 pandemic has spread awareness about health and hygiene but it still can’t prevent the transmission of vector-borne diseases. Like in COVID-19, the risky group comprises immuno-compromised individuals including pregnant women. The unavailability of potential vaccines against malaria, dengue virus, zika virus, and chikungunya virus, points on the importance of the vector control measures to reduce the epidemic transmission of diseases by the mosquito vectors such as *Anopheles stephensi* and *Aedes aegypti*^1,2^. The long term use of synthetic chemical insecticides have led to the micro-evolution of insecticide-resistant mosquitoes resulting in the constant occurrence of vector-borne disease cases despite the Integrated Vector Management (IVM) mediated controls in the endemic areas. This has led to the demand for vector control research in many sectors and also to formulate and evaluate the potential larvicidal formulations^3^. The research on the evaluation of the toxicity of different metal nanoparticles (NPs) for their potential use as larvicidal, pupicidal, and adulticidal agents in mosquito control have been well documented on monometallic NPs^4^. The increased one-pot synthesis of metal NPs using extracts of different biological origin like plant tissues, microbes, and fungi have provided an eco-friendly and cost-effective approach for the NPs synthesis^5–7^. The phytochemical extract of leaves of the plant *Citrus limon* is known to possess aromatic and medicinal importance with insect repellent properties. For instance, the aqueous leaf extract of *Citrus limon* is considered non-toxic and is also used in the preparation of green tea beverages due to its therapeutic activities. Its metabolomic profile shows the presence of active molecules of 26 different organic acids and their derivatives, 21 amino acids, and 13 sugars and sugar alcohols. Among these, the biological molecules like limonene, sabinene, citronellal, linalool, neral, geranial, ocimene, citronellol, and caryophyllene can also be purified and concentrated to be utilized in therapeutic, anti-microbial, insect-repellent, and natural pesticidal formulations^8^. The surface of biosynthesized nanoparticles is thus coated with the non-toxic by-products of the biological extract. The defects in the surface structure produced during the physical synthesis and the use of toxic reagents leading to the generation of hazardous by-products during the chemical synthesis of nanoparticles can be overcome by utilizing the natural bio-degradable extract and environmentally friendly approach of biological synthesis^9^. The non-toxic surface chemistry of biosynthesized nanoparticles can be further modified into biocompatible systems for their biomedical and therapeutic applications such as in bioimaging, drug delivery systems, theranostics, and other applications like biosynthesized nanocatalysts, anti-microbial food packaging materials, nanobiosensors, and disinfectants, etc.^10–13^. The vast applications of metal NPs into the environment cannot be denied and neither their nano-ecotoxicological impact on the organisms in the environment^14^. The biosynthesized monometallic nanoparticles with non-toxic surface functional molecules still possess different levels of toxicity based on the interaction of the type of metal nanoparticles with the organisms. The studies on toxicity evaluation of the noble nano-metals of silver, gold, and palladium, has shown that the silver nanoparticles are highly toxic due to their gradual oxidation and realize of highly reactive Ag + ions. This has led to the less toxic gold and palladium nanoparticles to gain interest in biomedical applications^15^. The monometallic NPs have known to demonstrate anti-bacterial properties and were tested positive for their anti-plasmodial, anti-cancer, pesticidal, and larvicidal activities^16–18^. Nevertheless, the combinatorial formulation of different combinations of the bimetallic nanoparticles (BNPs) are known to possess distinct properties as compared with their monometallic forms. The study on the different bimetallic NPs can show unique properties in terms of its synergistic or antagonistic combinatorial activity^19–21^. The studies on the evaluation of an eco-friendly extract mediated Au–Pd BNPs are known for its enhanced catalytic activity in comparison to their monometallic forms^22^. The availability of data on the use of monometallic Au NPs and Pd NPs in the area of catalysis, protein microsensors activity, imaging, and bioassay suggest the possibility for the use of the current biosynthesized Au–Pd BNPs in many areas in addition to its use in mosquito control^23–29^. The inadequate data in the field of toxic bioassay evaluation of Au–Pd BNPs against microbes, cell lines, pathogens parasites, and vectors of diseases indicate the future benefits of the novel findings of the current bimetallic nanoparticles. In the present study, we have utilized the non-toxic bioactive components of the aqueous leaf extract of *Citrus limon* as natural reducing and capping agents for the synthesis of Au–Pd BNPs. The bioassay was performed to evaluate the toxic bioassay of the synthesized Au–Pd BNPs against the invertebrate organisms such as mosquito larvae (*Anopheles stephensi* and *Aedes aegypti*) which serves as disease vectors for many parasitic and viral pathogens. The lethal concentration (LC_50_) obtained from the mosquito larvicidal bioassay was used to test the predation efficiency of the non-target invertebrate organisms (nymphs of dragonfly and damselfly) which serves as a natural predator on mosquito larvae, as an attempt to eco-toxic evaluation against invertebrate organisms.

## Results

The results of the present study reveal the physicochemical properties of the synthesized Au–Pd BNPs. The 10% aqueous leaf extract of *Citrus limon* showed no toxicity in the negative control which proves it’s non-toxic nature. The laboratory results for the efficacy of Au–Pd BNPs bioassay on the selected mosquito larvae provide an effective working lethal concentration (LC_50_) for 24 h, 48 h, and 72 h of exposure for each larval instar (I–IV). The effect of selected LC_50_ in the predation efficiency test showed mortality in the mosquito larvae population with no visible behavioral changes in the predatory nature of nymphs.

### Characterization

#### UV–visible (UV–VIS) spectroscopy

**Figure 1:**
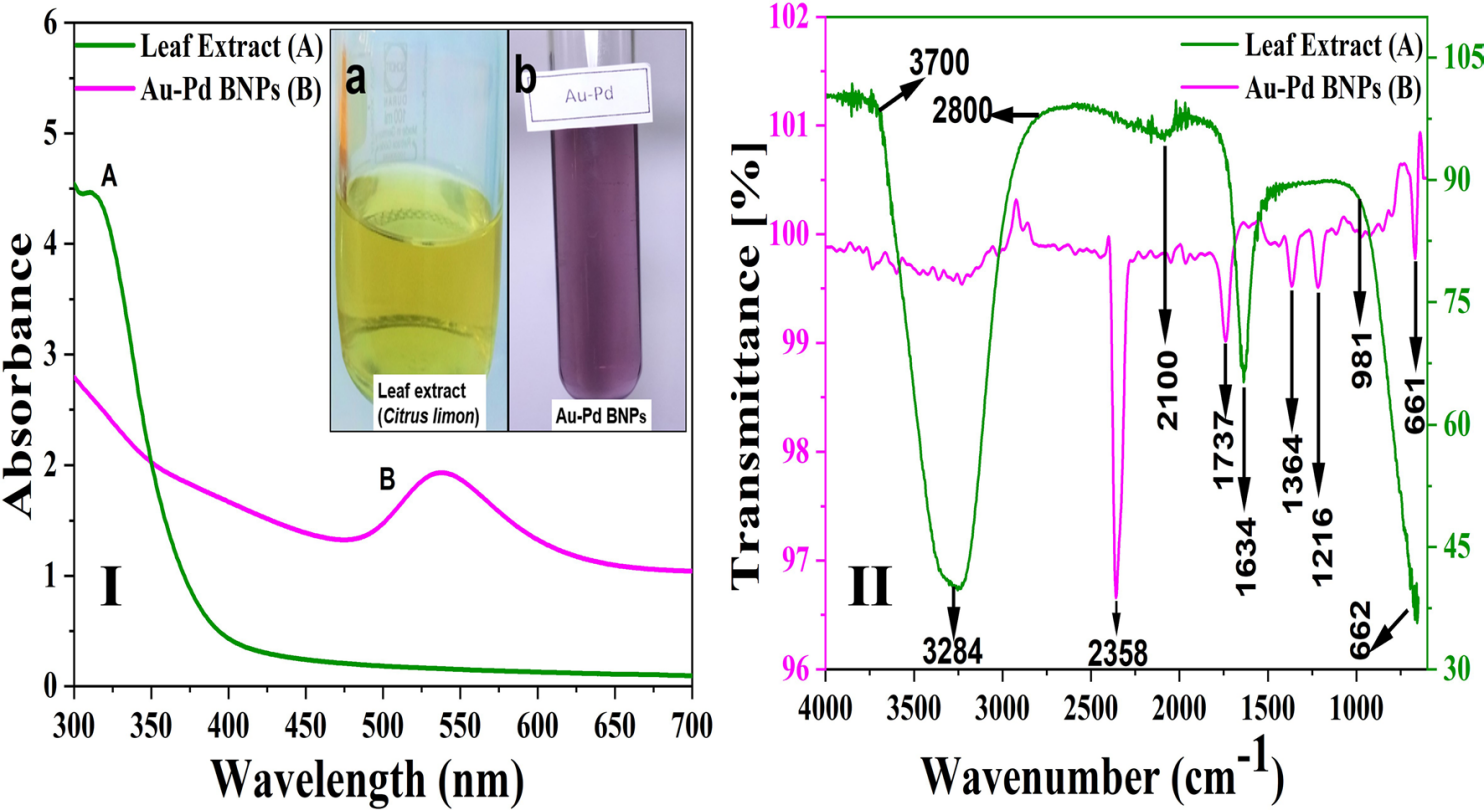
I) UV VIS spectroscopy analysis to obtain the absorbance spectra and II) FT-IR spectroscopy analysis to obtain the transmittance spectra, for: (**A**) the diluted (1:9) sample of 10% aqueous leaf extract of *Ctirus limon*, and (**B**) the diluted (1:10) sample of Au–Pd BNPs (2 mM) synthesized after the addition of 10% aqueous leaf extract of *Ctirus limon*; [Inset showing image of I (a) original sample of 10% aqueous leaf extract of *Ctirus limon* I (b) diluted sample (1:10) of 2 mM Au–Pd BNPs].

The absorbance spectra obtained for the 10% leaf extract of *Citrus limon* diluted (1:9) in DI water (Fig. 1I, A, a) showed the maximum absorption in the UV region around the wavelength (λ_max_) 300 nm ± 40 nm at 29 °C with the pH of 5.24. The surface plasmon resonance (SPR) band obtained for the diluted sample (1:10) of 2 mM Au–Pd (1:1) BNPs mediated by the prepared aqueous leaf extract of plant *Citrus limon* in 1:9 ratio (Fig. 1I, B, b), was observed in the visible region from 475 to 625 nm wavelength with λ_max_ around 537 nm at 29 °C with the pH of 2.23.

#### Fourier transform infrared (FT-IR) spectroscopy

Leaf extract: The FT-IR spectral analysis for the diluted sample (1:9) of the prepared 10% aqueous leaf extract of *Citrus limon* (Fig. 1II, A) showed a strong intensity peak at 3284 cm^−1^ with a broad transmittance band (3700–2800 cm^−1^), this corresponds to the intermolecular bonded O–H stretching vibrations of alcohols and carboxylic acids. This band also overlaps the functional group regions for N–H and C–H stretching vibrations of amines and alkynes, respectively. A weak band at 2100 cm^−1^ is due to C≡C stretching vibrations of alkynes and a medium band at 1634 cm^−1^ shows C=C stretching of alkene compound. The strong and broad band from 981 to 599 cm^−1^ shows the bending vibrations for the aromatic compounds with benzene ring structure and stretching vibrations for C–H group and the halogen groups such as C–Cl, C–Br, and C–I. Au–Pd BNPs: A transmittance band ranging from 3600 to 2800 cm^−1^ was significantly absent in the diluted sample (1:10) of Au–Pd BNPs (Fig. 1II, B). The high intensity peak at 2358 cm^−1^ has generated due to the O=C=O stretching of carbon dioxide present in the measuring environment. The weak band in Au–Pd BNPs at 1737 cm^−1^ corresponds to the C–H bending of the aromatic compounds. The weak bands at 1364 cm^−1^ and 1216 cm^−1^ in Au–Pd BNPs corresponds to the N–O stretching of nitro compounds and C–N stretching of amines, respectively. The weak band from 981 to 599 cm^−1^ is highly reduced in comparison to the leaf extract.

#### Transmission electron microscopy (TEM)

**Figure 2:**
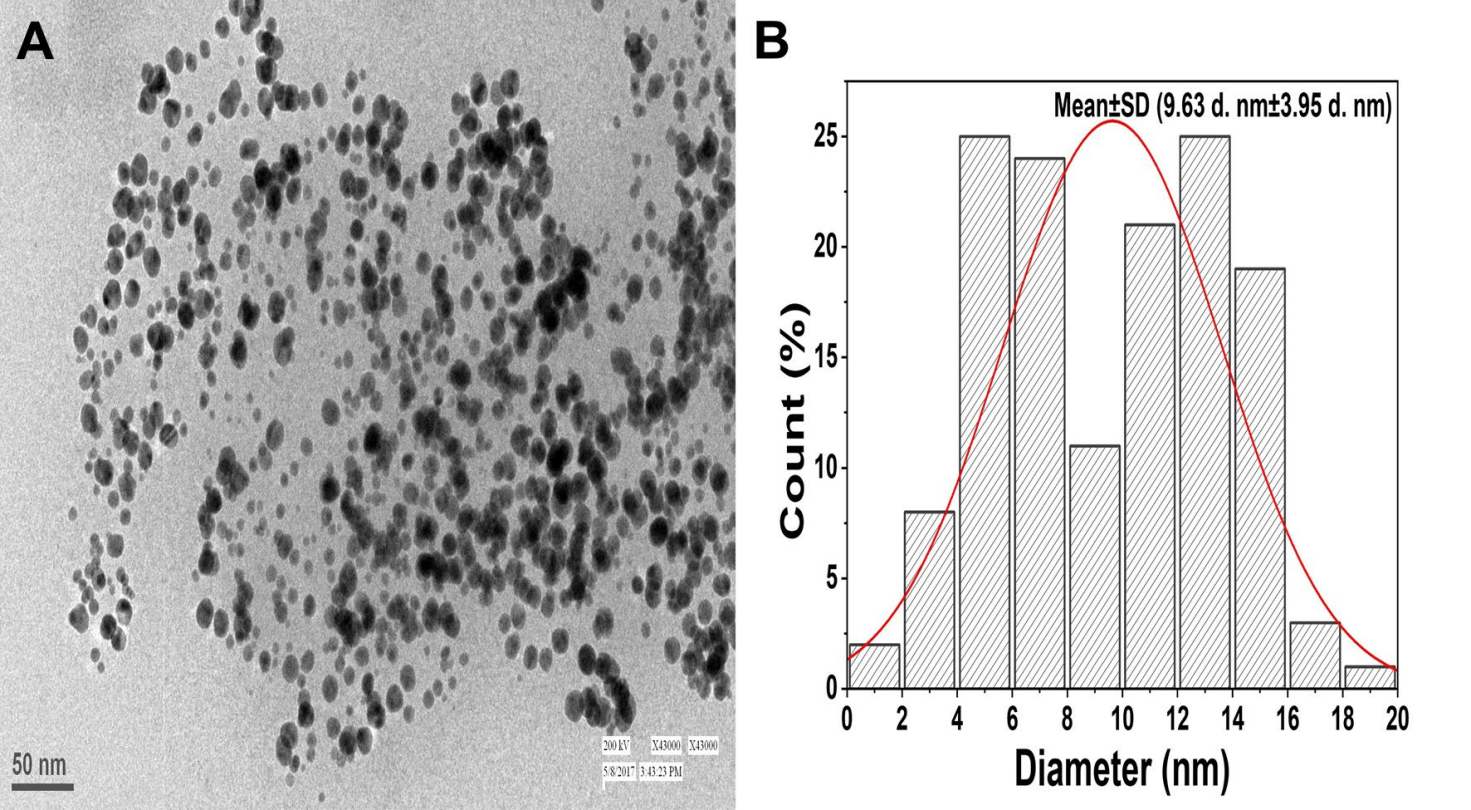
(**A**) TEM micrograph generated for the original sample of the 2 mM Au–Pd BNPs synthesized after the addition of 10% aqueous leaf extract of *Citrus limon*; (**B**) Particle size distribution histogram plot generated from the size measurement data obtained by the ImageJ analysis of the nanoparticles observed in the TEM micrograph (**A**).

The visual analysis of the micrographs obtained for synthesized Au–Pd BNPs sample shows the presence of the homogenous spherical shaped nanoparticles (Fig. 2A). The particle size distribution bins generated for the selected TEM micrograph (Fig. 2A) shows the presence of nanoparticles in the size range from ~ 1.5 to ~ 18.5 d. nm and the distribution curve shows the mean particle size (± SD) of ~ 9.63 d. nm (± 3.95 d. nm) (Fig. 2B). This data suggests the synthesis of the polydisperse spherical nanoparticles.

#### Energy dispersive x-ray (EDX) spectroscopy of SEM view

**Figure 3:**
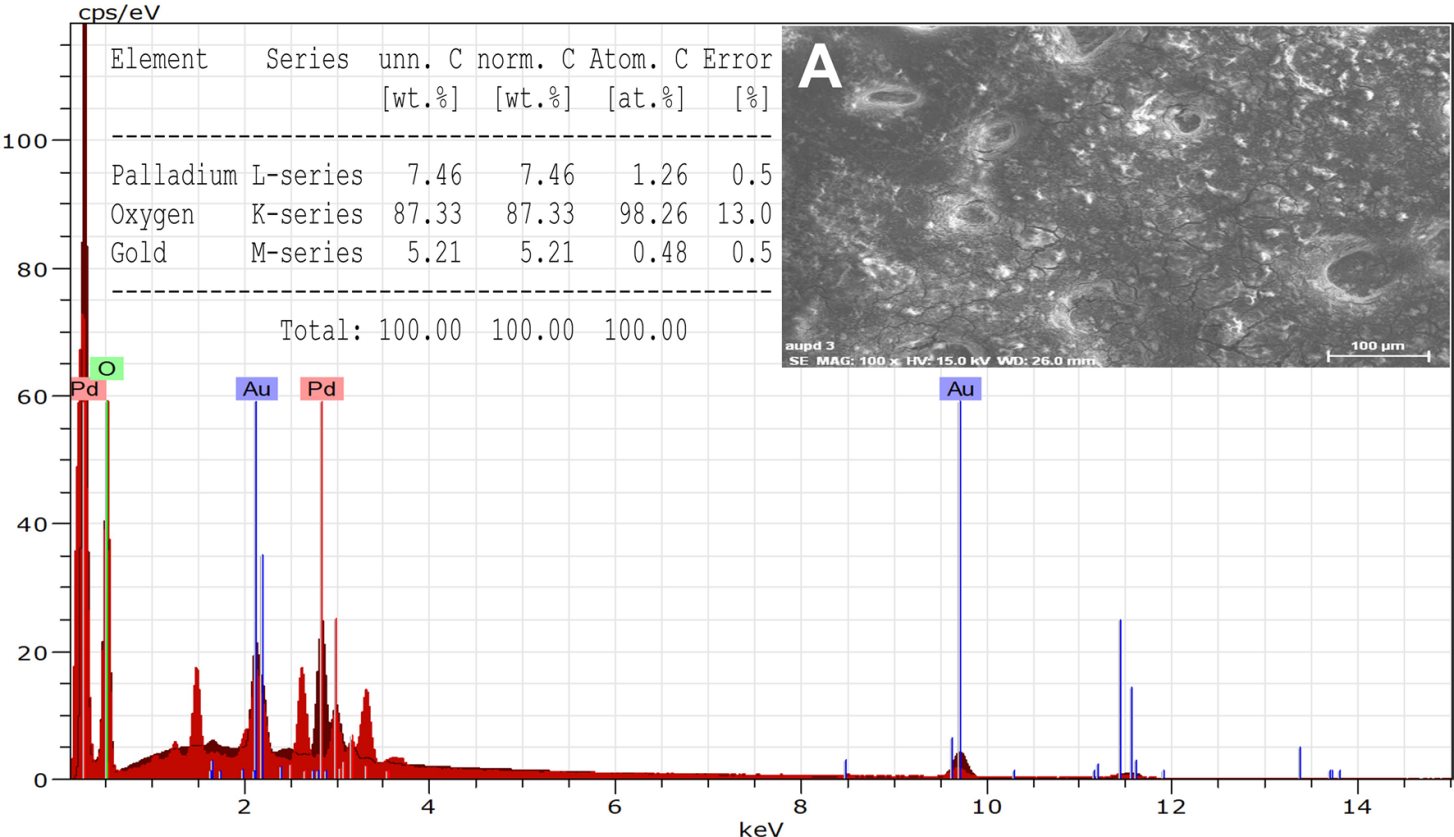
EDX Analysis for original sample of the synthesized 2 mM Au–Pd BNPs using the 10% aqueous leaf extract of *Ctirus limon* [Inset showing A) SEM micrograph of Au–Pd BNPs, and the EDX table for elements analysis results].

The EDX spectrum for Au–Pd BNPs (Fig. 3) was generated for the entire SEM view (Fig. 3A). The elemental composition confirmed to be pure with an absence of other metal contaminants. The element % for Au, Pd, and oxygen is of 5.21, 7.46, and 87.33, respectively resulting from their respective atomic % of 0.48, 1.26, and 98.26.

#### XRD

**Figure 4:**
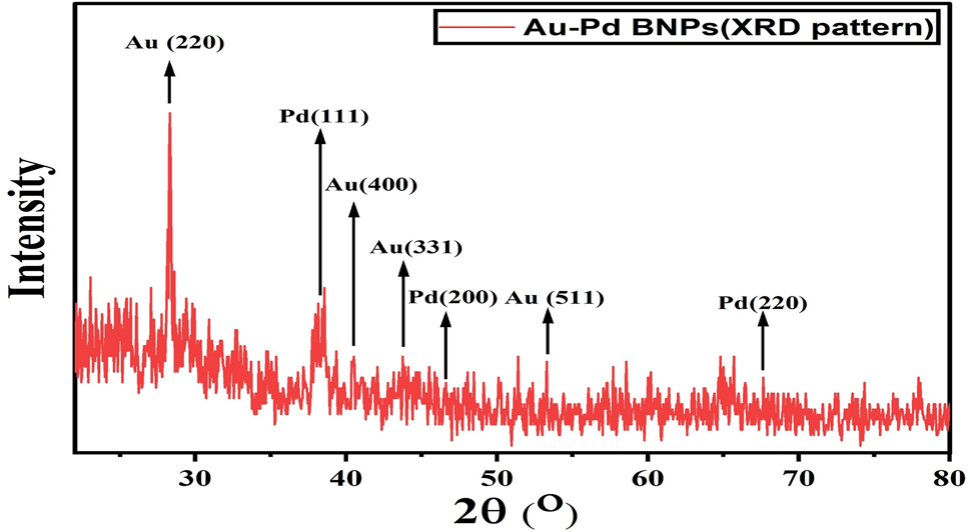
XRD pattern generated for the original sample of the 2 mM Au–Pd BNPs using 10% aqueous leaf extract of the plant *Ctirus limon*.

The XRD diffraction pattern for Au–Pd BNPs (Fig. 4) shows peaks at 28.3°, 34.7°, 40.6°, and 53.3° of 2θ angle, corresponding to Au content of the sample (JCPDS no. 01-1172) with lattice constants (220), (311), (400), and (511), respectively. The peaks at 38.5°, 46.6°, and 67.65° corresponding to Pd content of the sample (JCPDS no. 65-2867) with lattice constants (111), (220), and (311), respectively.

#### DLS and ZP

**Figure 5:**
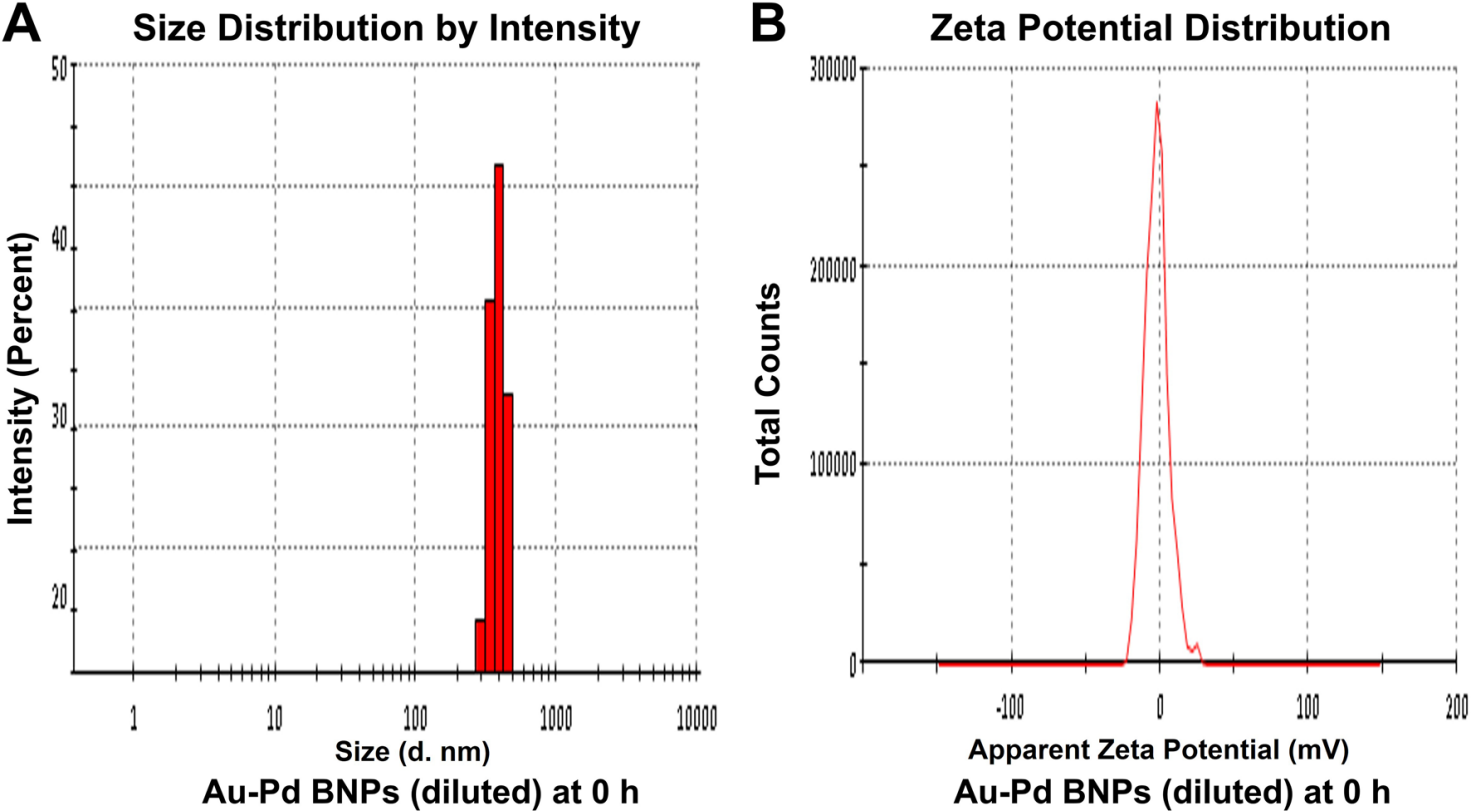
(**A**) DLS analysis and (**B**) Zeta Potential analysis, of the diluted (1:10) sample of 2 mM synthesized Au–Pd BNPs using 10% aqueous leaf extract of the plant *Citrus limon* at 0 h of synthesis.

**Figure 6:**
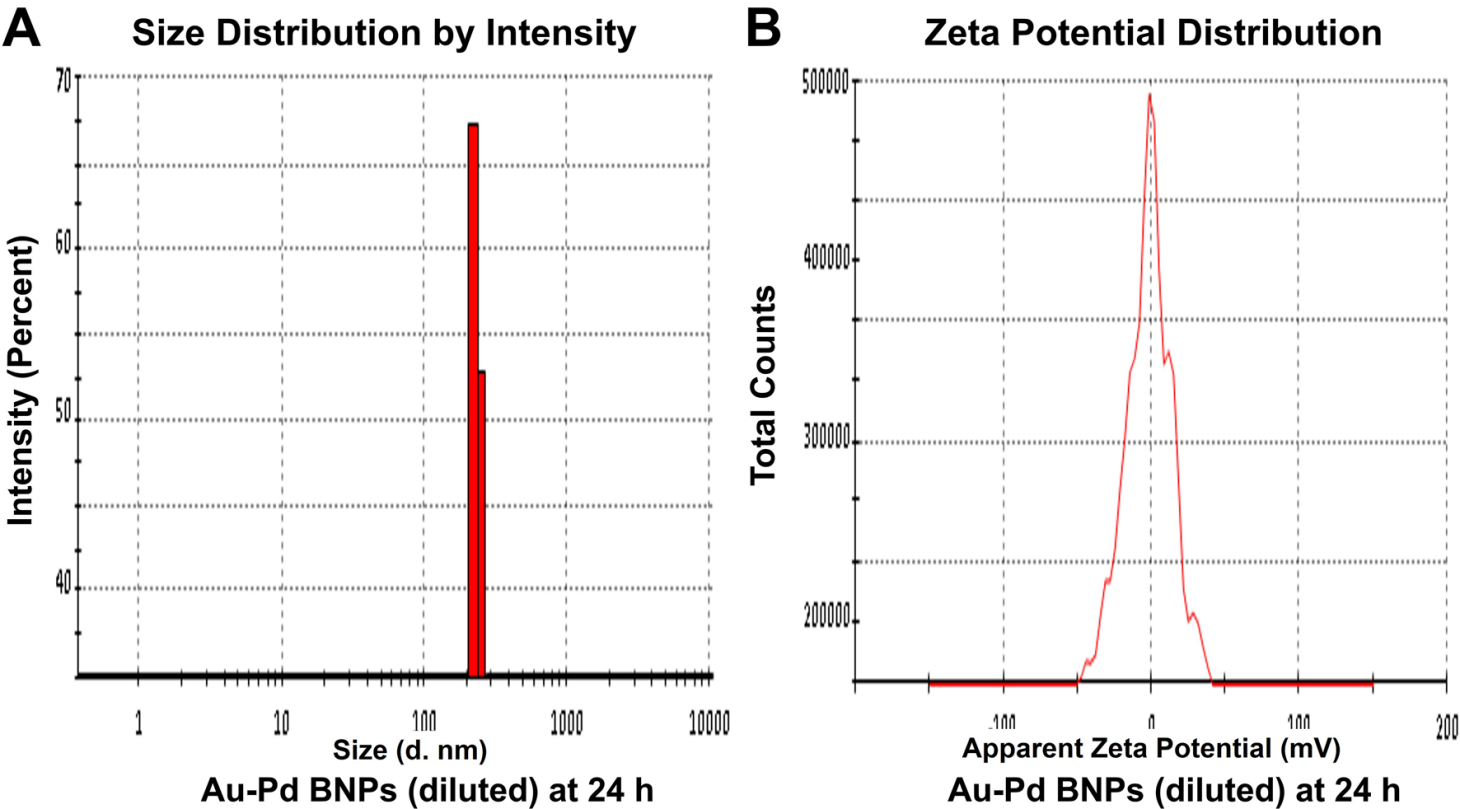
(**A**) DLS analysis and (**B**) Zeta Potential analysis, of the diluted (1:10) sample of 2 mM synthesized Au–Pd BNPs using 10% aqueous leaf extract of the plant *Citrus limon* at 24 h of synthesis.

**Figure 7:**
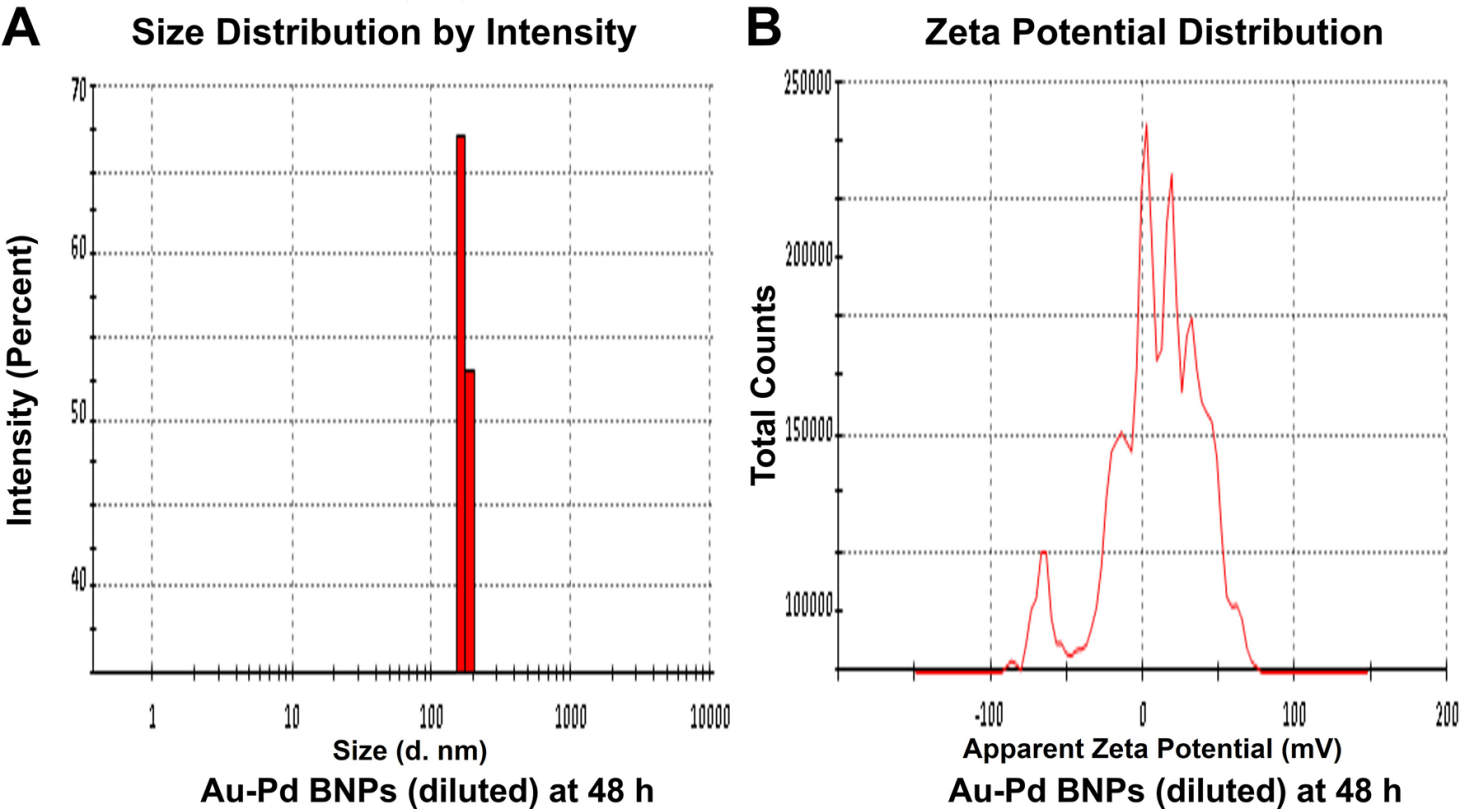
(**A**) DLS analysis and (**B**) Zeta Potential analysis, of the diluted (1:10) sample of 2 mM synthesized Au–Pd BNPs using 10% aqueous leaf extract of the plant *Citrus limon* at 48 h of synthesis.

**Figure 8:**
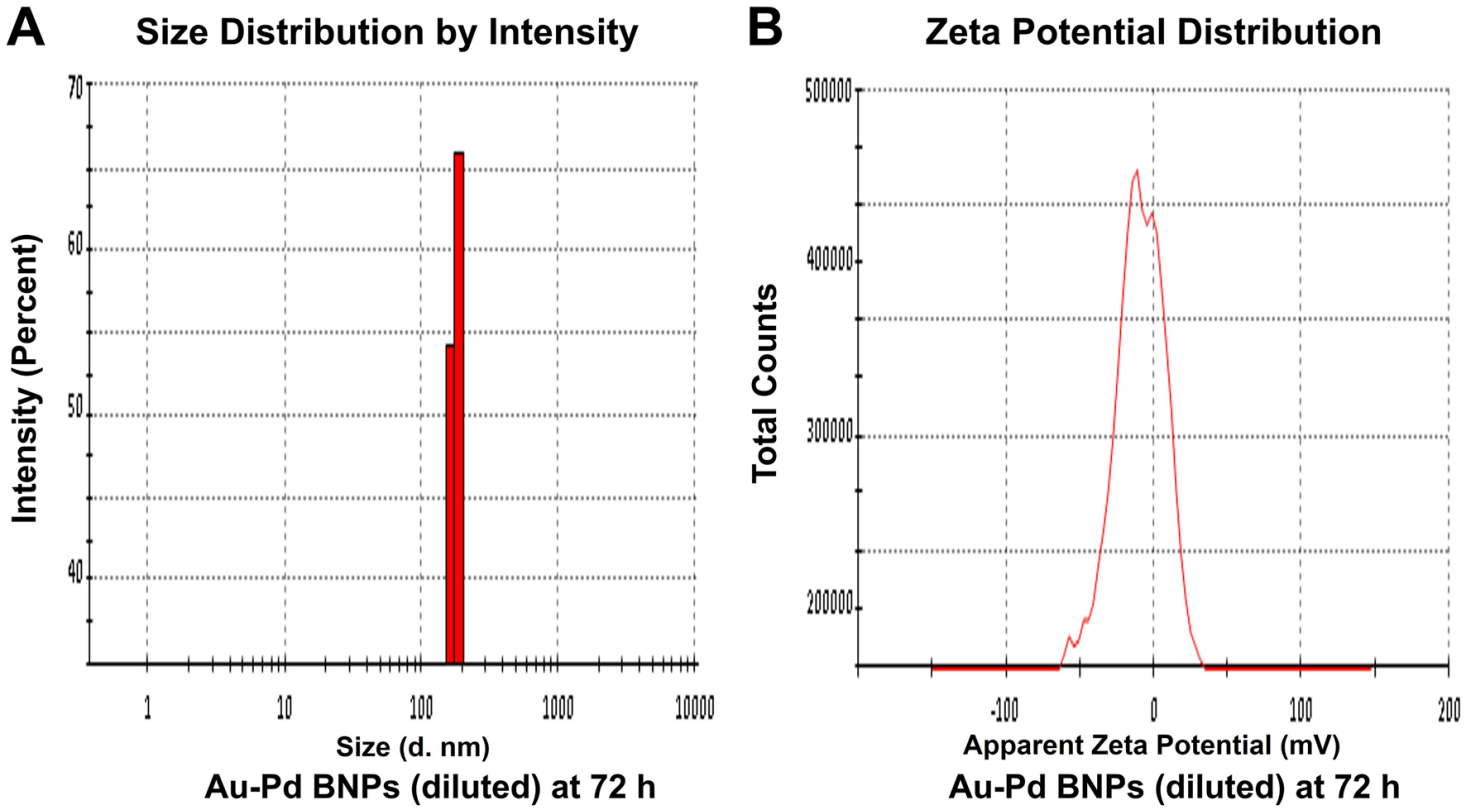
(**A**) DLS analysis and (**B**) Zeta Potential analysis, of the diluted (1:10) sample of 2 mM synthesized Au–Pd BNPs using 10% aqueous leaf extract of the plant *Citrus limon* at 72 h of synthesis.

The zeta size analysis (Figs. 5A, 6, 7, 8A) of the diluted sample (1:10) of synthesized Au–Pd BNPs (2 mM) showed a Z-Average (d. nm) of 823.3, 2885, 3340, and 3795, with the peak (size in d. nm) observed at 389.5, 232.6, 173.5, and 180.2, at 0 h (Fig. 5A), 24 h (Fig. 6A), 48 h (Fig. 7A), and 72 h (Fig. 8A) of synthesis, respectively. The PdI value obtained at 0 h was 0.744, and PdI value of 1 was observed for the samples at 24 h, 48 h, and 72 h of synthesis. The zeta-potential (mV) (Figs. 5B, 6, 7, 8B) obtained for Au–Pd BNPs sample at 0 h (Fig. 5B), 24 h (Fig. 6B), 48 h (Fig. 7B), and 72 h (Fig. 8B) was − 2.16, − 8.66, − 5.28, and − 9.30, respectively. The mean peaks observed for the zeta-potential (mV) at 0 h of synthesis was − 2.32 and 24.1, at 24 h of synthesis was − 7.09, 14.1, and 29.3, at 48 h of synthesis was 38.2, 1.59, and 17.4, and at 72 h of synthesis was − 18.5, 5.19, and − 56.8.

### Au–Pd BNPs bioassay

**Figure 9:**
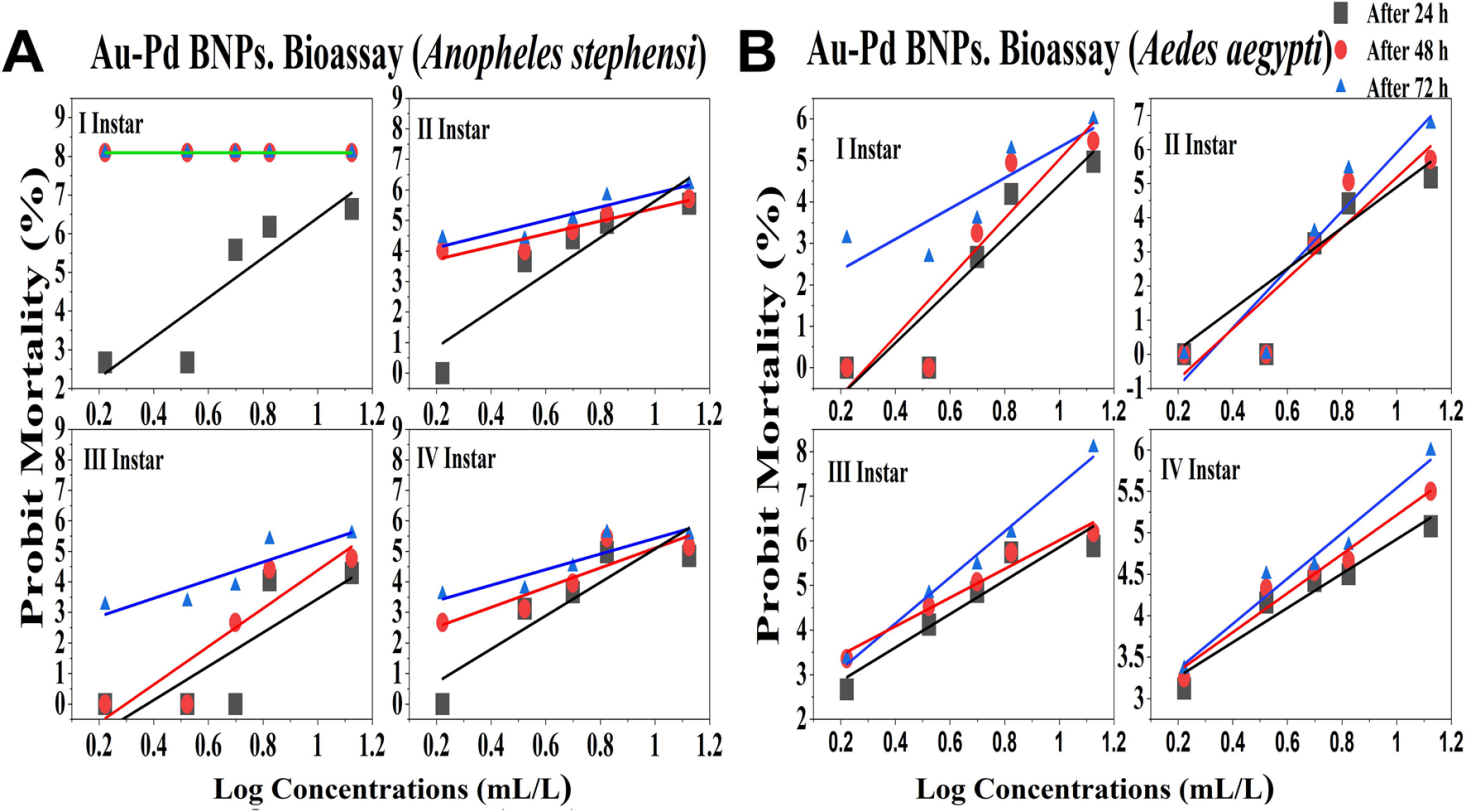
Probit regression plots generated from the Au–Pd BNPs bioassay data for I–IV instar larvae after 24 h, 48 h and 72 h of exposure of the Au–Pd BNPs test concentrations for: (**A**) larvae of *Anopheles stephensi* mosquito; (**B**) larvae of *Aedes aegypti* mosquito.

**Figure 10:**
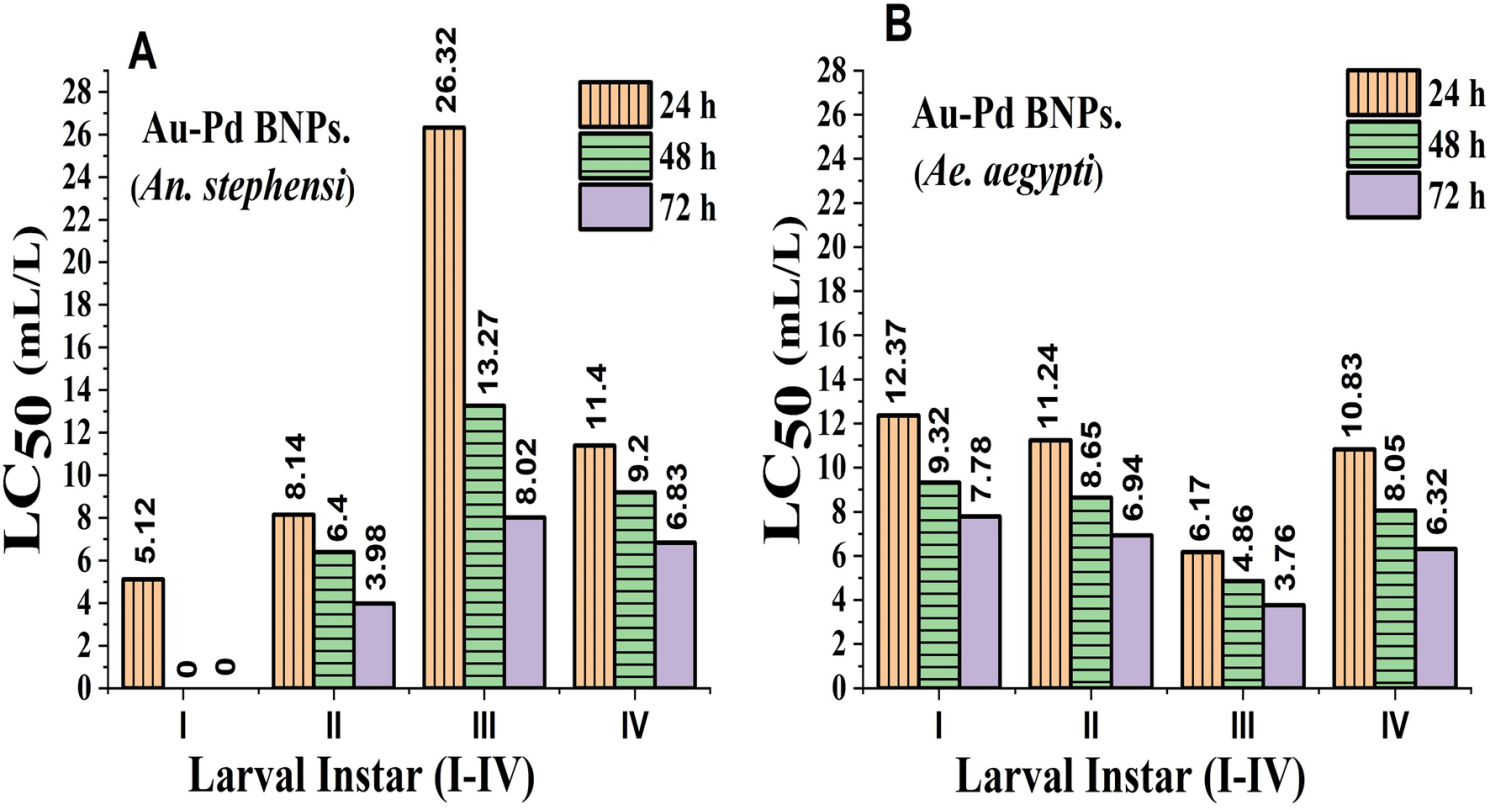
LC_50_ observed in the test concentrations of Au–Pd BNPs bioassay against I-IV instar larval stages of: (**A**) larvae of *Anopheles stephensi* mosquito; (**B**) larvae of *Aedes aegypti* mosquito.

The positive control showed 100% mortality within 24 h of exposure. No mortality in the negative control of leaf extract and distilled water was observed after 24 h, 48 h and 72 h of exposure. The probit analysis plots were generated (Fig. 9A,B) from the corrected % mortality calculated from the efficacy data of Au–Pd BNPs test concentrations. For Au–Pd BNPs bioassay (*An. stephensi*), the LC_50_ for I instar larvae was observed in 5.12 mL/L test concentration at 24 h, while 100% mortality was observed in 1.67 mL/L (LC_99_) test concentration at 48 h and 72 h of exposure. The LC_50_ for II instar larvae was observed in the test concentration of 8.14 mL/L, 6.40 mL/L, and 3.98 mL/L, at 24 h, 48 h, and 72 h of exposure, respectively. The LC_50_ for III instar larvae was observed in the test concentration of 26.32 mL/L, 13.27 mL/L, and 8.02 mL/L at 24 h, 48 h, and 72 h of exposure, respectively. The LC_50_ for IV instar larvae was observed in the test concentration of 11.40 mL/L, 9.20 mL/L and 6.83 mL/L at 24 h, 48 h, and 72 h of exposure, respectively. For Au–Pd BNPs bioassay (*Ae. aegypti*), the LC_50_ for I instar larvae was observed in the test concentration of 12.37 mL/L, 9.32 mL/L, and 7.78 mL/L at 24 h, 48 h, and 72 h of exposure, respectively. The LC_50_ for II instar larvae was observed in the test concentration of 11.24 mL/L, 8.65 mL/L, and 6.94 mL/L, at 24 h, 48 h, and 72 h of exposure, respectively. The LC_50_ for III instar larvae was observed in the test concentration of 6.17 mL/L, 4.86 mL/L, and 3.76 mL/L, at 24 h, 48 h, and 72 h of exposure, respectively. The LC_50_ for IV instar larvae was observed in the test concentration of 10.83 mL/L, 8.05 mL/L, and 6.32 mL/L, at 24 h, 48 h, and 72 h of exposure, respectively. The reduced chi-square test (χ^2^) at 1–3 degrees of freedom (*d.f.)* under the probability of 0.05 (5% level of significance) was performed for all stages of both mosquito species. The significant (S) χ^2^ values representing a bad-fit with no association between the test concentrations and the observed mortality was obtained for the Au–Pd BNPs test concentrations for III and IV instar larvae of *Anopheles stephensi* at 24 h of exposure, and for I and II instar larvae of *Aedes aegypti* at 48 h of exposure, this can be due to the type II error. While non-significant (NS) χ^2^ values which predict goodness of the fit and shows an association between the test concentrations and observed mortality, was obtained for the rest of the Au–Pd BNPs tests concentrations at 24 h, 48 h, and 72 h of exposure. The graphical representation (Fig. 10A,B) of the LC_50_ values obtained for each larval instar (I–IV) of both mosquito species shows a comparative conclusive representation on the time-dependent change in the LC_50_ values.

### Predation efficiency test

**Figure 11:**
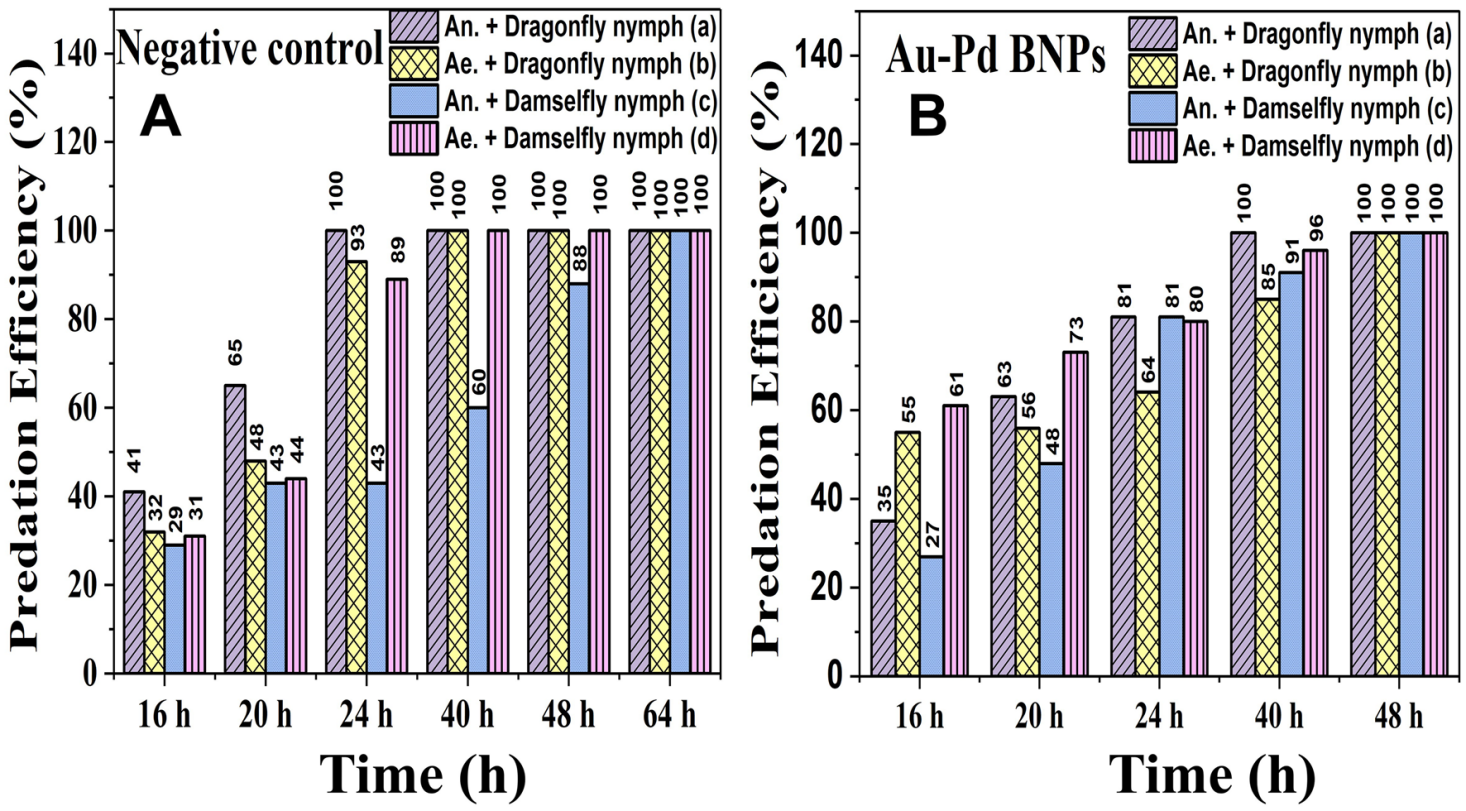
(**A**) Negative Control and (**B**) Au–Pd BNPs Concentration to test the predation efficiency of the (a) Dragonfly nymphs with III instar larvae of *Anopheles stephensi* (An.), (b) Dragonfly nymphs with III instar larvae of *Aedes aegypti* (Ae.), (c) Damselfly nymphs with III instar larvae of *Anopheles stephensi*, and (d) Damselfly nymphs with III instar larvae of *Aedes aegypti* (Ae.).

The negative control (Fig. 11A) showed the normal feeding behavior of the nymphs in the laboratory set up. The observations were made until all the larvae were predated in the test. The complete predation was observed till 64 h of the test set up. No predator mortality was observed. In Fig. 11A, a, c we can see that the dragonfly nymphs (Fig. 11A, a) are voracious feeders on the larvae of *An. stephensi* (An.) in comparison with damselfly nymphs (Fig. 11A, c). Owing to the surface feeding behavior of *An. stephensi* larvae, the predation activity of damselfly nymphs (Fig. 11A, c) appeared to be slow as compared to all the other groups where 100% predation was achieved at 40 h (Fig. 11A, a, b, d). The bottom-feeding behavior of *Ae. aegypti* (Ae.) larvae made it more susceptible to predation by both predators.

The predation efficiency test at the selected test concentration of Au–Pd BNPs (6.67 mL/L) was done to evaluate predatory behavior of nymphs in the laboratory set up. The test concentration was selected from the observations made in mosquito larvae bioassay. In Fig. 11B, 100% predation at 48 h in all groups was observed as compared to the incomplete predation (88%) in (damselfly and *An. stephensi*) negative control at 48 h (Fig. 11A, c). Comparatively reduced predation in Au–Pd BNPs test (Fig. 11B, a, b, d) is observed for the test groups containing, Dragonfly nymph and *An. stephensi*, Dragonfly nymph and *Ae. aegypti*, *and* Damselfly nymphs, and *Ae. aegypti* at 24 h as compared to 100% predation at 24 h in the respective negative control (Fig. 11A, a, b, c). On the contrary, an increased (91%) predation at 24 h in Au–Pd BNPs test group of Damselfly nymphs and *An. stephensi* (Fig. 11B, c) was observed as compared to 60% predation after 24 h in the respective negative control (Fig. 11A, c).

## Discussion

The characterization results of the unprocessed original samples of the prepared 10% aqueous leaf extract of *Citrus limon* and synthesized Au–Pd BNPs (2 mM) provided insight into its unique properties which can be studied for further alteration to improvise its use in other applications. In the current study, the absorbance of leaf extract samples in the UV region (Fig. 1I, A, a) shows a characteristic photons absorption spectrum facilitated by the bonds and rings of the biological macromolecules present in the selected leaf extract sample^8^. The localized surface plasmon resonance (SPR) band observed in the BNPs sample (Fig. 1I, B, b) is a result of the collective oscillations of the free electron in the conduction band of the nanoparticles^30^. The SPR band for Au NPs is usually observed in the wavelength range from 490 to 600 nm, while no SPR band for Pd NPs is observed due to the absence of free electrons (surface plasmons) in its outer shell^23,31^. The FT-IR analysis results for the current study suggest the presence of new surface functional groups in the Au–Pd BNPs sample (Fig. 1II, B) as compared to the leaf extract sample (Fig. 1II, A). The N–O stretching of nitro compounds and C-N stretching of amines is clearly observed in the transmittance spectra for the Au–Pd BNPs samples (Fig. 1II, B), which were absent in the leaf extract sample (Fig. 1II, A). The considerable utilization of the O–H groups (3600–2800 cm^−1^) and aromatic compounds (981–599 cm^−1^) of the leaf extract in the synthesis of Au–Pd BNPs (Fig. 1II, B), suggests the formation of the Au–Pd oxide BNPs^31^. This information can be utilized for further stabilization and surface modification of the Au–Pd BNPs samples though the use of surface compatible biogenic ligand molecules, polymers, or surfactants, for its use in biomedical applications. The resulting shape and size can be a combinatorial effect of the two metals and the eco-friendly approach of 10% aqueous leaf extract (*Citrus limon*) mediated synthesis. Metal combinations of Au–Pd BNPs, can also provide variable results in size and shape for each type of extract and method of synthesis used. The native activity of the extract used for the reduction and capping of the metal ions is also responsible for the stability of synthesized nanoparticles. Agglomeration and nanocluster formation can be a by-product of the ingredients present in the type of extract used^32,33^. The NPs often settle down to the bottom owing to their nucleation and growth and due to the interaction of the surface molecules on the nanoparticles which can be overcome with the use of polymeric stabilizers and sonication. The EDX analysis results showed a high concentration of oxygen in the composition of Au–Pd BNPs (Fig. 3). This indicates the synthesis of gold–palladium oxide BNPs which supports the results of FT-IR analysis (Fig. 1II, B). The XRD diffractogram (Fig. 4) shows the semi-crystalline nature of the samples and the presence of high noise confirms the leaf extract based synthesis of Au–Pd BNPs^22^. The ZP (mV) deals with the surface charge of the particles, particularly with the electrostatic repulsive forces. However, insight on the stability of any nano-formulation can be assessed with both electrostatic repulsive forces and van der Waals attractive forces. For a solution having particles with weak van der Waals attractive forces, the colloid stability can be assessed by the mild electrostatic repulsive forces as reflected by the low ZP (± 10 to ± 15 mV). Therefore, a stable nanoparticles sample may have a low ZP since it only deals to provide the indicative information of the nature of surface charge i.e. positive or negative. The samples prepared in DI water often show 2–10 nm larger zeta size than the actual size in DLS results. The colored samples containing metal nanoparticles often show absorbance at the wavelength of laser (633 nm) which can lead to the estimation of the incorrect size of the particles. The metal nanoparticles having shapes other than spherical cannot be estimated by DLS. Hence, the determination of physicochemical properties of the metal nanoparticles using DLS and ZP results are often compared with the FT-IR spectroscopy and TEM results to draw conclusions on the surface functional groups and, shape and size of the nanoparticles, respectively. However, the visual results of TEM and intensity-based results of DLS are fundamentally different but the former provides an actual size and shape of the electron-dense metal nanoparticles, unlike the latter one which shows a comparatively bigger size based on the hydrodynamics of the particles present in the sample^34^. The scattering plots of DLS are often inclined towards detecting data with combined high-intensity scattering, provides biased results. In polydisperse samples, the occurrence of masking of low-intensity scattering of the small-sized particles by high-intensity scattering of large-sized particles is evident^35^. The current DLS analysis of the diluted samples (1:10) of 2 mM Au–Pd BNPs (Figs. 5A, 6, 7, 8A), shows the hydrodynamic size of the particles, which includes both Au–Pd BNPs and the surface capping agents of the leaf extract. These biogenic surface functional groups (Fig. 1II, B) can aid nanoparticles to agglomerate in the Au–Pd BNPs sample over time. This data clearly suggests that the rate of agglomeration in the Au–Pd BNPs solution can be due to the interaction between the denatured macromolecules of the leaf extract and the surface functional groups of the Au–Pd BNPs. The actual hydrodynamic size of the unaltered samples of synthesized Au–Pd BNPs shows the greater particle size (Z-Average) in comparison to the actual size of the metal nanoparticles which is enclosed in the leaf extract capping agents. The actual size of the metal nanoparticles can be determined by the ImageJ analysis of the obtained TEM micrograph ^33^. In the present bioassay results, the selected concentration range was effectively toxic against the larvae of the selected mosquito larvae species and the data can be used for the selection of the lower concentrations for time-based efficacy for safe environmental applications. The positive control shows high larvicidal efficacy at the selected concentration. The absence of mortality after 72 h of exposure in the leaf extract and distilled water negative control shows, the non-toxic nature of aqueous leaf extract and survivability in the absence of food, respectively, for the selected mosquito larvae bioassay. The concentration was mentioned in mL/L to provide an accurate measurement of the test. From the obtained efficacy results we can see that the value of LC_50_ concentration decreases with time for each stage (Fig. 10A, B). Hence, the time-dependent efficacy evaluation can also be adopted for the minimal LC_50_ selection. The 100% mortality in Au–Pd BNPs bioassay was observed against I instar of *An. stephensi* mosquito larvae but not for I instar larvae of *Ae. aegypti* mosquito after 24 h. From this finding, we can say that the mode of action of BNPs concentrations has different effects on both species owing to their physical and biological complexity. The current study of the predation efficiency test shows that the initial predation in Au–Pd BNPs test (Fig. 11B, a–d) was slow at 24 h but increased after 24 h of Au–Pd BNPs exposure as compared to the negative control (Fig. 11A, a–d). This suggests the increase in predation due to the availability of slow-moving, inactive or morbid larvae as compared to normal quick-moving larvae. No mortality in the predator (nymphs) group at the selected test concentration till 72 h was observed. The test nymphs were kept in the laboratory for 72 h after the test to observe the post-exposure effects on the predator which showed normal behavior and moulting.

The absence of literature for the efficacy of Au–Pd BNPs bioassay against mosquito larvae and its effect on the predatory behavior of non-target aquatic invertebrate insects is due to the novelty of the current study. However, sufficient studies are available on the synthesis of Au–Pd BNPs from different biological extracts such as ascorbic acid and *Cacumen platycladi* leaf extract mediated Au–Pd BNPs (~ 40 nm) showed an efficient catalytic ability for the oxidation of benzyl alcohol to benzaldehyde^32^. The mono and bi-metallic nanoparticles of Au and Pd synthesized using the flower extract of *Lantana camara* plant showed enhanced catalytic activity of Au–Pd BNPs as compare to the individual Au NPs and Pd NPs for the reduction of hazardous borohydride dye^22^. The fenugreek plant polysaccharide mediated Au–Pd BNPs also showed heterogeneous catalytic hydrogenation of toxic 4-nitrophenol to the environmental friendly 4-aminophenol compounds^36^. *Euphorbia condylocarpa* root extract mediated Au–Pd BNPs can catalyze the ligand-free Suzuki and Heck coupling reactions in water with the unaltered catalytic activity even after several repeated cycles^37^. The novel biosynthesis of metal nanoparticles can also, be studied further by utilizing the purified active phytochemical compounds instead of using the whole crude leaf extract. This type of study can provide information on the effect of different types of active phytochemical compounds in the eco-friendly and non-toxic formation and stabilization of metal nanoparticles. This approach can enhance the surface functional properties of synthesized nanoparticles as it prevents the accumulation of the diverse organic ingredients responsible for nanoparticles agglomeration in the original solution. Based on the findings of current results, further research in the use of Au–Pd BNPs can be evaluated for environmental or biomedical applications. The present study on the eco-friendly synthesis of Au–Pd BNPs based mosquito larvicidal formulation shows the eco-toxicological findings of the metals nanoparticles alone. During the synthesis of the current Au–Pd BNPs, the structures of the active ingredients of non-toxic leaf extract were modified in the formation of the nanoparticles in the original solution as seen in FT-IR results (Fig. 1II, A,B). Therefore, we suggest that an effective mosquito larvicidal formulation can be prepared by combining the plant-based active larvicidal ingredients with the synthesized nanoparticles. Further, the bioassay of such formulation can be evaluated for the selection of an effective LC_50_. Lastly, we suggest further research in the scientific community to test the antibacterial activity of Au–Pd BNPs in the bacterial biofilms along with the anti-viral bioassays for its use in disinfectants.

## Methods

### Material

HAuCl_4_.3H_2_O and PdCl_2_ (HiMedia), triple deionized (DI) water, HCl (Merk), Temper (EC) (Temephos), Whatman filter paper no.-1.

### Collection of Leaves, Mosquito larvae and Nymphs of Non-Target organisms

The fresh leaves of *Citrus limon* (Linnaeus) Burm. f. were collected from the Botanical Garden of Dayalbagh Educational Institute (DEI), Agra, India (27°, 10′ N, 78° 05′ E). The larvae of *Anopheles stephensi* (Liston) and *Aedes aegypti* (Linnaeus) mosquitoes, and the nymphs of Dragonfly and Damselfly were also collected from the surrounding water bodies in DEI campus. The online identification keys from Walter Reed Biosystematics Unit (WRBU) were followed for mosquito species identification^38^. Pictorial keys from the Indian Biodiversity Portal were used to identify the native adult Dragonfly (*Bradinopyga geminata* (Rambur, 1842)) and Damselfly (*Ischnura senegalensis* (Rambur, 1842)) in the surrounding environment^39,40^.

### Preparation of aqueous leaf extract of plant Citrus limon

The preparation of aqueous leaf extract and nanoparticles synthesis was performed using the one-pot synthesis method. The 10% aqueous leaf extract of plant *Citrus limon* was prepared with 10 gm of clean green finely chopped leaves into 100 mL of deionized water. The leaf broth was heated at 65–70 °C for 1 h. The filtered broth (10%) was used on the same day and stored at 4 °C for further use.

### Synthesis of Au–Pd BNPs

To synthesize Au–Pd BNPs, the prepared leaf extract was added into the 2 mM solution of the metal salt solution in 1:9 ratio. The 100 mL of the 2 mM final solution of Au–Pd BNPs consist of 1 mM of each metal salts such as PdCl_2_ (17 mg in 45 ml DI water + 5 ml leaf extract) and HAuCl_4_.3H_2_O (39 mg in 45 ml DI water + 5 mL leaf extract) in 90 mL of deionized water. The Au–Pd BNPs synthesis was divided into three steps: First, the 1 mM PdCl_2_ salt was first dissolved in 0.5 mL HCl followed by the addition of 45 mL of DI water and 5 mL of the prepared leaf extract in drop-wise manner and the preparation was kept in dark. Second, the 1 mM HAuCl_4_.3H_2_O dissolved in 45 mL of DI water and heated at 120–180 °C until bubbles are seen, followed by the drop-wise addition of 5 mL of prepared leaf extract. Third, both pre-formed nanoparticles solutions containing Au and Pd salts and leaf extract were mixed at the initial stage of visible color change in the heated HAuCl_4_.3H_2_O and leaf extract solution and the final solution was stirred for 5 min at 450 rpm. The final, 2 mM bimetallic solution (100 mL) containing pre-formed NPs were left in dark for 24 h. At this stage, the nucleation and growth of nanoparticles were allowed at room temperature (29 °C). The synthesis was performed in the covered glasswares. After 24 h, the Au–Pd BNPs were stored at 4 °C.

### Characterization

The prepared aqueous leaf extract (10%) and Au–Pd BNPs (2 mM), were characterized after 48 h ± 2 h of synthesis. The samples of both leaf extract and Au–Pd BNPs were diluted in DI water in 1:9 and 1:10 ratio, respectively, to be analyzed by UV–Visible (UV VIS) spectroscopy (Hitachi U-3900 spectrophotometer) and Fourier transform infrared (FT-IR) spectroscopy (Bruker TENSOR 37 FTIR). A blank containing DI water was set for the baseline correction for both techniques. The UV VIS spectroscopy was performed to obtain absorbance spectra and surface plasmon resonance (SPR) bands in the wavelength ranging from 300 to 700 nm, with 600 nm/min scan speed, 2 nm slit width, and 5.0 mm path length. The FT-IR data was used to analyze the modified functional groups present in Au–Pd BNPs samples as compared to the original functional groups in the leaf extract sample. The transmittance peaks were recorded in the mid-infrared region ranging from 599 to 3996 cm^−1^ in wavenumber. The pH of the synthesized 2 mM Au–Pd BNPs sample and 10% *Citrus limon* leaf extract was also measured at 29 °C. The undiluted sample of synthesized 2 mM Au–Pd BNPs was used for Transmission Electron Microscopy (Technai G 20 (FEI) TEM), Scanning Electron Microscopy (SEM – Zeiss EV040) with Energy Dispersive X-ray spectroscopy (PANalytical X’pert PRO), and X-ray Diffractometer (D8 ADVANCE Bruker) analysis. The TEM visualization of the dried Au–Pd BNPs sample over a formvar coated copper grids was done to generate the micrographs for the visual analysis of synthesized nanoparticles. The different locations of the same grid were observed to generate the micrographs of the test BNPs sample. In addition to the visual interpretation of the shape and size of the nanoparticles relative to the nanoscale, the selected TEM micrograph was also analyzed for the particle size distribution in diameter nm (d. nm). The particle area of the nanoparticles visible in the selected TEM micrograph was obtained using the ImageJ software and the diameter was calculated to generate the histogram displaying the size distribution bins. The distribution curve was generated from the histogram plot to obtain the value of the mean particle size ± standard deviation. The SEM–EDX analysis was used to assess the elemental composition of the dried Au–Pd BNPs sample over a carbon tape on the SEM stub. The crystallinity of the dried Au–Pd BNPs sample over the 1 cm x 1 cm glass slide was achieved by XRD analysis. The dynamic light scattering (DLS) and zeta potential (ZP) (Malvern Zetasizer Nano ZS90) was observed for the diluted Au–Pd BNPs (2 mM) samples in 1:10 ratio in the DI water, at 25 °C. The hydrodynamic size was measured through DLS (zeta size) and its surface electrical charge was analyzed through ZP at 0 h, 24 h, 48 h, and 72 h of synthesis for the stored Au–Pd BNPs sample (diluted) at a temperature variation of 29 °C ± 4 °C.

### Au–Pd BNPs bioassay

The toxicity of the synthesized Au–Pd BNPs was evaluated on the mosquito larvae of *Anopheles stephensi* and *Aedes aegypti*. The guidelines for the laboratory testing of mosquito larvicides by WHO (2005) was followed for the determination of LC_50_^41^. The Au–Pd BNPs bioassay was performed on I, II, III, and IV instar of the larvae of selected mosquito species. The collected larvae were washed twice with distilled water and segregated according to the larval stages. The source water was incubated in a bio-incubator at 28 °C for the extraction of the newly hatched I instar. The selection of the test concentrations was done after the general laboratory survey of a wide range of test concentrations against the IV instar. The specific range of test concentrations was selected on the basis of 50% of observed larval mortality after 24 h. The test concentrations of 0.25, 0.5, 0.75, 1, and 2 mL of Au–Pd BNPs in 150 mL of distilled water containing 25 larvae were set up in three replicates for I, II, III, and IV instar. The selected test concentrations were equivalent to the strength of 1.67, 3.34, 5, 6.67, and 13.34 in mL/L of Au–Pd BNPs as such used in the data analysis for the bioassay. A triplicate of each control containing 0.2% Temephos (EC) for positive control and two types of negative controls containing 13.34 mL/L of 10% leaf extract of *Citrus limon* and another with plain distilled water were observed for mortality. The mortality was observed at 24 h, 48 h, and 72 h for Au–Pd BNPs bioassay and negative controls, and at 4 h, 8 h, and 24 h for positive control.

### Data analysis for Au–Pd bioassay

The dose–response relationships in the Au–Pd BNPs test concentrations were analyzed for the calculation of LC_50_ through Probit Analysis (Finney 1952)^42^. The obtained mortality (%) data were subjected to corrected mortality (%) if 5–20% mortality in the negative control is observed (Abbott, 1925)^43^. The corrected mortality (%) was then transformed into respective probit values using the probit table (Finney 1948)^44^. The comparative regression graphs were plotted to generate the probit equations (ŷ-value) for the calculation of LC_50_ or LC_99_ values. The LC_50_ values were also represented graphically to draw conclusive remarks on time and concentration based comparative data obtained for the I-IV larval instars of both *Anopheles stephensi* and *Aedes aegypti* mosquito species.

### Predation efficiency test

The Predation Efficiency (P.E.) test was evaluated on the selected invertebrate predatory nymphs of dragonfly and damselfly which are generally found in the local water bodies along with the mosquito larvae^45^. The nymphs measuring ~ 1 inch in length were selected for the predation efficiency test^46^. The selected nymphs were fed on the III instar larval stage of each *Anopheles stephensi* and *Aedes aegypti* mosquito species in both test and negative control. The negative control containing 25, III instar larvae, and 1 nymph in plain distilled water was installed in triplicates. The test was evaluated for an effective lethal concentration as selected from the results of III instar larval mortality of each mosquito species in Au–Pd BNPs bioassay. The observations were made at 16 h, 20 h, 24 h, 40 h, 48 h, 64 h, and 72 h of Au–Pd BNPs exposure in the bioassay and in the negative control. The predation efficiency test (%) was calculated using the Eq. (1): ((No. of consumed mosquito larvae / No. of predators) /Total no. of mosquito larvae) × 100 ^46^.

## Conclusions

In the present study, we report the synthesis Au–Pd bimetallic nanoparticles using the non-toxic and eco-friendly aqueous leaf extract of plant *Citrus limon*. The bioassay of the synthesized Au–Pd BNPs showed larvicidal efficacies at different concentrations but no mortality in non-target organisms was seen in the test. The time-based analysis of the bioassay results shows that the toxic concentrations (LC_50_) were found to be decreased with the increase in time (h). Hence, the selection of an effective dose can be reduced based on the life-cycle of the target and non-target organism. The physical, chemical, and optical characterization data of *Citrus limon* mediated Au–Pd BNPs can be utilized for its use in different applications. Hereby, we conclude the current study provides an evaluation of the concentration and time-based bioassay of Au–Pd BNPs against the larval instars (I-IV) of the selected mosquito species. Its effects on the predation efficiency of non-target aquatic nymphs of the selected insects were also evaluated in order to conserve the natural biological control agents of mosquito in the environment.

## Supplementary information

Supplementary Information.
