# Supplementary material for: Laboratory analysis of Au–Pd bimetallic nanoparticles synthesized with Citrus limon leaf extract and its efficacy on mosquito larvae and non-target organisms
Source: Sci Rep. 2020 Dec 10;10:21610. doi: 10.1038/s41598-020-78662-y (PMC7728787; doi:10.1038/s41598-020-78662-y)
Supplement: Supplementary file 1 — Supplementary Information. [file 41598_2020_78662_MOESM1_ESM.pdf]

Title: Laboratory Analysis of Au-Pd Bimetallic Nanoparticles Synthesized with *Citrus limon* leaf extract and its Efficacy on Mosquito larvae and Non-Target Organisms

Ref: Submission ID 451f4d21-c0f8-4023-9a7f-50b5ef26c74b

Savy Panamkuttiyiel Minal[1,*], and Soam Prakash[1]

[1]Advance Parasitology and Vector Control Nano-Biotechnology Laboratory, Department of Zoology, Dayalbagh Educational Institute (Deemed University), Agra, India - 282005
[*]savypanamkuttiyielminal@gmail.com; soamprakash@dei.ac.in

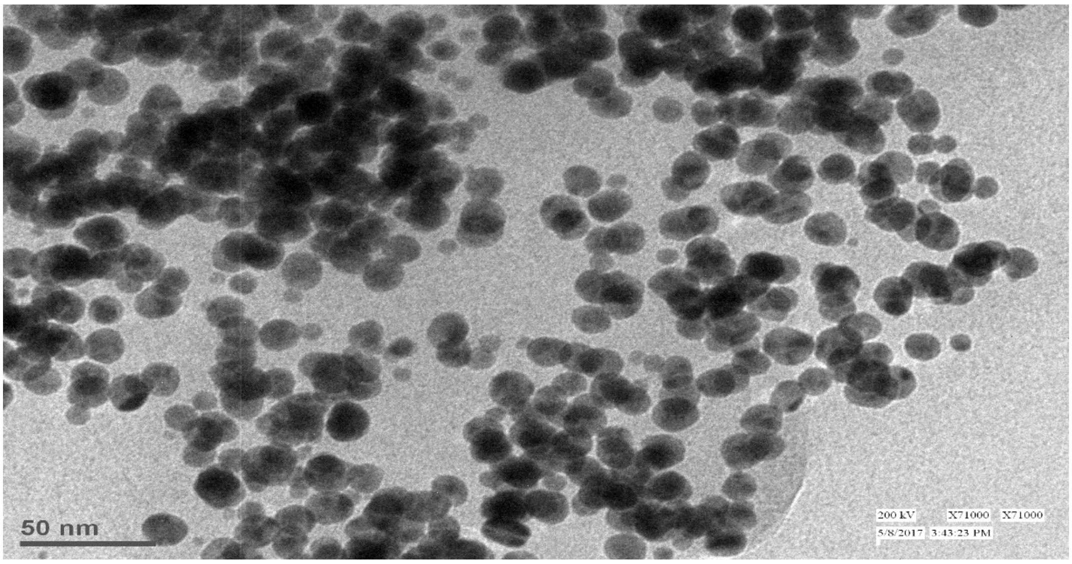

Figure 2. i. TEM micrograph of the undiluted sample of the synthesized Au-Pd BNPs

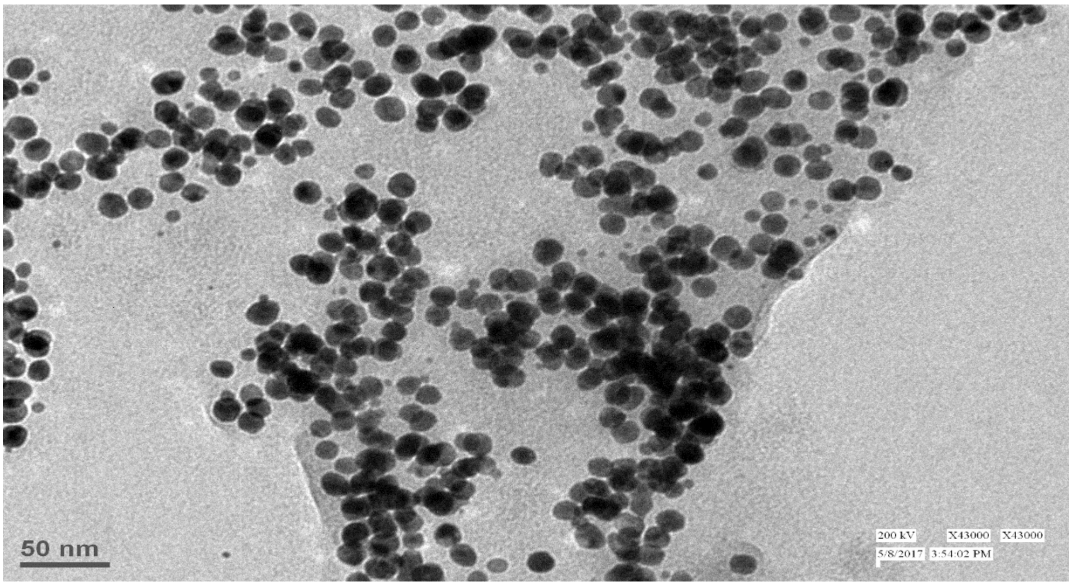

Figure 2. ii. TEM micrograph of the undiluted sample of the synthesized Au-Pd BNPs

A. Au-Pd BNPs Bioassay against *Anopheles stephensi*									
**Instar**	**Time**	**Probit equation (ŷ) (log_{10})**	R^2	**SD**	**SE**	χ^2	**LC_{50} (mL/L)**	**95% Fiducial CI (mL/L)**	
								**Lower**	**Upper**
**I**	**24**	ŷ = 4.422*x*+1.863	0.86	0.20	0.05	0.14 (NS)	**5.12**	**4.15**	**6.33**
**II**	**24**	ŷ = 3.031*x*+2.240	0.98	0.33	0.07	0.67 (NS)	**8.14**	**5.94**	**11.16**
	**48**	ŷ = 2.038*x*+3.357	0.89	0.48	0.09	0.67 (NS)	**6.40**	**4.23**	**9.68**
	**72**	ŷ = 2.166*x*+3.699	0.86	0.45	0.09	0.63 (NS)	**3.98**	**2.695**	**5.89**
**III**	**24**	ŷ = 0.780*x*+3.416	0.84	1.28	0.28	-	**26.32**	**14.88**	**46.55**
	**48**	ŷ = 3.580*x*+0.980	0.89	0.25	0.07	0.01 (NS)	**13.27**	**9.67**	**18.21**
	**72**	ŷ = 2.823*x*+2.447	0.81	0.34	0.07	0.17 (NS)	**8.02**	**5.81**	**11.08**
**IV**	**24**	ŷ = 3.027*x*+1.800	0.84	0.32	0.08	0.03 (S)	**11.40**	**8.06**	**16.13**
	**48**	ŷ = 3.056*x*+2.054	0.78	0.32	0.07	0.05 (NS)	**9.20**	**6.68**	**12.68**
	**72**	ŷ = 2.479*x*+2.932	0.82	0.39	0.08	0.40 (NS)	**6.83**	**4.80**	**9.71**
**B. Au-Pd BNPs Bioassay against *Aedes aegypti***									
**I**	**24**	ŷ = 4.405*x*+0.188	0.95	0.21	0.06	0.07 (NS)	**12.37**	**9.33**	**16.39**
	**48**	ŷ = 4.220*x*+0.909	0.93	0.22	0.06	0.01 (S)	**9.32**	**7.24**	**12.00**
	**72**	ŷ = 2.829*x*+2.480	0.64	0.27	0.06	0.06 (NS)	**7.78**	**5.91**	**10.23**
**II**	**24**	ŷ = 4.038*x*+0.758	0.97	0.24	0.06	0.14 (NS)	**11.24**	**8.45**	**14.94**
	**48**	ŷ = 4.746*x*+0.552	0.94	0.20	0.05	0.02 (S)	**8.65**	**6.87**	**10.90**
	**72**	ŷ = 6.628*x*-0.577	0.98	0.15	0.04	0.10 (NS)	**6.94**	**5.79**	**8.33**
**III**	**24**	ŷ = 3.449*x*+2.274	0.95	0.28	0.06	0.90 (NS)	**6.17**	**4.71**	**8.09**
	**48**	ŷ = 3.123*x*+2.857	0.96	0.31	0.06	0.97 (NS)	**4.86**	**3.63**	**6.49**
	**72**	ŷ = 4.538*x*+2.388	0.99	0.22	0.05	0.99 (NS)	**3.76**	**2.99**	**4.74**
**IV**	**24**	ŷ = 2.083 x+2.846	0.93	0.47	0.10	0.82 (NS)	**10.83**	**6.99**	**16.76**
	**48**	ŷ = 2.341 x+2.880	0.96	0.42	0.08	0.87 (NS)	**8.05**	**5.49**	**11.80**
	**72**	ŷ = 2.695 x+2.841	0.96	0.37	0.07	0.84 (NS)	**6.32**	**4.53**	**8.82**

Table 1. Statistical analysis of the percent mortality data for the calculation of the LC_{50} at 24 h, 48 h, and 72 h of exposure of Au-Pd BNPs test concentration in the bioassay against I-IV instar larval stages of, A) *Anopheles stephensi* mosquito, and B) *Aedes aegypti* mosquito
